# Patient-reported outcome measures to assess mental and physical health status, functionality, and quality of life in patients with major depression or schizophrenia

**DOI:** 10.1186/s41687-024-00804-x

**Published:** 2025-01-09

**Authors:** Luis San, Belen Arranz, Carlota Romans, Berta García, Marta Coromina, Sonia Ortiz, Miriam Vilaplana, Víctor Soto, Ruth Villaescusa, Joan Alvaros

**Affiliations:** 1 Parc Sanitari Sant Joan de Déu, General Hospital, C/ Doctor Antoni Pujadas 42, Sant Boi de Llobregat, Barcelona, E-08830 Spain; 2 Centro de Investigación Biomédica en Red de Salud Mental (CIBERSAM), Barcelona, Spain

**Keywords:** Patient-reported outcome measures (PROMS), Adult patients, Major depression, Schizophrenia

## Abstract

**Background:**

Patient-reported outcomes measures (PROMs) are standardized self-administered tools that assess the patient’s opinion on the level of health, quality of life, and disability among other aspects. The objective of this study was to gather information on physical and mental health in patients with major mental illness using PROMs.

**Methods:**

This was an observational, naturalistic, prospective study carried out in adult stabilized outpatients attended at nine Adult Mental Health Centers in Barcelona, Spain. All participants had a confirmed diagnosis of major depression disorder or schizophrenia (DSM-5) and were currently on drug treatment. Participants (*n* = 508) self-completed a baseline questionnaire for clinical data and PROMs scales (PHQ-19, SF-12, and WHODAS 2.0) at baseline and 9 months thereafter (*n* = 482).

**Results:**

Mean (SD) age was 50.9 (13.2) years, and 83% of patients lived with their families. Although 93.9% of patients recognized having a mental illness, 15.7% did not know their diagnosis. When asked if they considered that during the last year their treatment had offered some type of improvement, 83.9% answered affirmatively. Patients reported that their degree of adherence to treatment was high (77%) and most of them (80%) believed the medication had a beneficial effect. Depressed patients showed both at baseline and at follow up significantly more depressive symptoms than the group with schizophrenia. In the schizophrenia group, a statistically significant improvement in depressive symptoms was noted at 9 months follow-up. We did not find significant differences within or between groups in quality of life (SF-12 scores) obtained at baseline and after 9 months of follow-up Both at baseline and at follow-up, patients with depression reported a significantly higher degree of disability (WHODAS scores) than those with schizophrenia.

**Conclusions:**

PROMs can be used in real-world conditions to assess severity of disease, quality of life, and disability in major depression and schizophrenia. The present results are relevant for both patients and clinicians.

## Introduction

Health care systems have increasingly focused on the need of individuals following a patient-centered approach. This proposal ensures that clinical decisions are respectful and responsive to patients’ unique preferences and values [1]. Patient-reported outcomes measures (PROMs) and patient-reported experience measures (PREMs) have become standardized instruments for assessing quality of care, success of treatment, satisfaction with care or overall attention received in terms of which are important to patients [2].

Health outcomes using PROMs have been extensively used, mostly limited to common conditions and those with a high burden of disease (e.g. cancers, cardiovascular disorders) [3]. In this respect, the International Consortium for Health Outcomes Measures (ICHOM), a non-profit institution that promotes an international collaborative project to determine indicators of health outcomes, has made efforts to develop a set of easily accessible patient-reported instruments for monitoring the outcomes that matter most to patients, including depression & anxiety and psychotic disorders in the category of mental health [4]. However, in the field of mental health there still are some open questions like what kind of tool could better capture the patient´s opinion, or what variables should be considered in the mental health assessment, or how to integrate evaluation using PROMs in routine clinical practice. So far, several systematic reviews and metaanalysis have included PROMs in patients diagnosed with several mental disorders [5–9]. A Cochrane systematic review and meta-analysis of 12 studies including 3696 participants with common mental disorders [10] did not find sufficient evidence to support the use of routine outcome monitoring using PROMs in common mental disorders for improving patient outcomes or management. Several factors, such as the psychiatric disorder, the low patient completion rates, the lack of insight, the length of follow up or the effect of psychotropic drugs might undermine the PROMs value and utilization in clinical practice.

The present study was conducted to assess the usefulness of PROMs in daily practice for assessing physical and mental health of outpatients with major depressive disorder or schizophrenia followed for 9 months. The objectives of the study were: (1) to gather information about the self-reported physical and mental health states of patients diagnosed with major depressive disorder or schizophrenia and to perform a comparison between both groups; (2) to integrate the voice of the patient as part of the care process; and (3) to empower patients by actively seeking and incorporating their feedback and insights, helping attending psychiatrists to enhance decision-making regarding diagnosis and treatment strategies.

## Materials and methods

### Study design and participants

This prospective observational and naturalistic study recruited participants of nine Adult Mental Health Centers integrated into the Parc Sanitari Sant Joan de Déu complex in Barcelona, Spain. Eligible patients were adults over 18 years of age who met criteria for diagnosis of major depressive disorder or schizophrenia according to the Diagnostic and Statistical Manual of Mental Disorders, fifth edition, (DSM-5) [11]. The Structured Clinical Interview for DSM-5 (SCID-5) was used for clinical diagnosis. It was administered by a clinical psychiatrist acquainted with the DSM-5 classification and diagnostic criteria [11]. All patients attended visits in the participating outpatient centers between November 2021 and November 2022. A minimum follow-up of 9 months after recruitment was also an inclusion criterion. These two diagnoses of depression [12] and schizophrenia [13] were selected because they reflect both the most prevalent and severe mental illnesses in stabilized outpatients with capacity to complete the study questionnaires attended in our practice. Patients with a prior diagnosis of low IQ or with reading issues were not included as they would have had difficulties answering a self-report questionnaire.

This study was conducted in accordance with the guidelines of the Declaration of Helsinki and was approved by Sant Joan de Déu Research Foundation (reference number PIC-126-21, approval date June 23, 2021). The coordinating psychiatrist (L.S.) with the support of the research team (psychiatrists, psychologists, and nurses) provided full information to each participant and collected the signed informed consent forms. Details of the informed consent included agreement on digital recording for questionnaires and scales and usage for scientific research after anonymization, as well as data collection and verification of original data in accordance with the usual requirements and/or policy of the promoter (Sant Joan de Déu Research Foundation). The consent form also included information about the fact that clinical scales would be administered at baseline and after 9 months.

### Study procedures and data collection

The main data source of this study was the information obtained from the study questionnaires that patients self-completed on the PROMs platform (digitally or paper form), the visualization of which was accessible to both researchers and patients. The study questionnaires fulfilled by patients at baseline (with data referred to the last year) and 9 months thereafter were considered original data and were selected to gather unified information on different aspects that were considered relevant to patients with major depression or schizophrenia. Data recorded included sociodemographic characteristics, clinical and treatment-related variables, patient’s recovery, patient’s attitude towards medication, comorbidities (other organic diseases), and adverse events.

According to recommendations of ICHOM, Spanish validated versions of the following scales were also administered: the Patient Health Questionnaire (PHQ-9) [14, 15], the Short Form Health Survey (SF-12) [16, 17], and the World Health Organization Disability Assessment Schedule 2.0 (WHODAS 2.0) [18, 19].

The PHQ-9 is a multipurpose instrument for screening, diagnosing, monitoring and measuring the severity of depression. The PHQ-9 incorporates DSM-IV depression diagnostic criteria with other leading major depressive symptoms into a brief self-report tool. It is a self-administered version of the PRIME-MD diagnostic instrument for common mental disorders. The PHQ-9 is the depression module, which scores each of the 9 DSM-IV criteria as “0” (not at all) to “3” (nearly every day).

The SF-12 is a health-related quality of life (HRQoL) questionnaire consisting of 12 questions that measure 8 health domains to assess physical and mental health. Physical health-related domains include general health, physical functioning, role physical, and bodily pain. The SF-12 is one of the most widely used instruments for assessing self-reported health-related quality of life.

The adult self-administered version of WHODAS 2.0 is a 36-item instrument that assesses disability across six domains, including understanding and communicating, getting around, self-care/hygiene, getting along-interacting with people, life activities/domestic responsibilities (i.e., household, work, and/or school activities), and participation in society. Results from WHODAS were calculated according to the “simple scoring” method, in which the scores from each item are simply added up without recoding or collapsing response categories. As a result, the simple sum of the scores of the items across all domains constitutes a statistic that is sufficient to describe the degree of functional limitations.

### Data analysis

Estimation of the sample size was not performed since accepting or rejecting any previously established hypothesis was not the purpose of the study. However, about 500 patients diagnosed with major depressive disorder or schizophrenia fulfilling the inclusion criteria were expected to be recruited by the nine Adult Mental Health Centers involved in the study. Univariate descriptive analysis was performed on all primary objective variables. Continuous variables are described using standard measures of centralized tendency and dispersion (mean, median, standard deviation, quartiles and eventually truncated means and percentiles). Qualitative, binary and ordinal variables will be analyzed using absolute and relative frequencies. Crossovers between variables are presented using absolute and relative frequencies and eventually cumulative frequencies will be used in ordinal variables. The distribution of categorical variables between the groups of patients with depression and those with schizophrenia was analyzed with the chi-square test or the Fisher’s exact test for categorical data, and the Student’s t test or the Mann-Whitney U test for quantitative data according to conditions of application. One way ANOVA with repeated measures was used to test differences between groups over time. The correlation of the scores of the different scales was analyzed with the Pearson’s rank correlation coefficient. Statistical significance was set at *p* < 0.05.

## Results

**Fig. 1:**
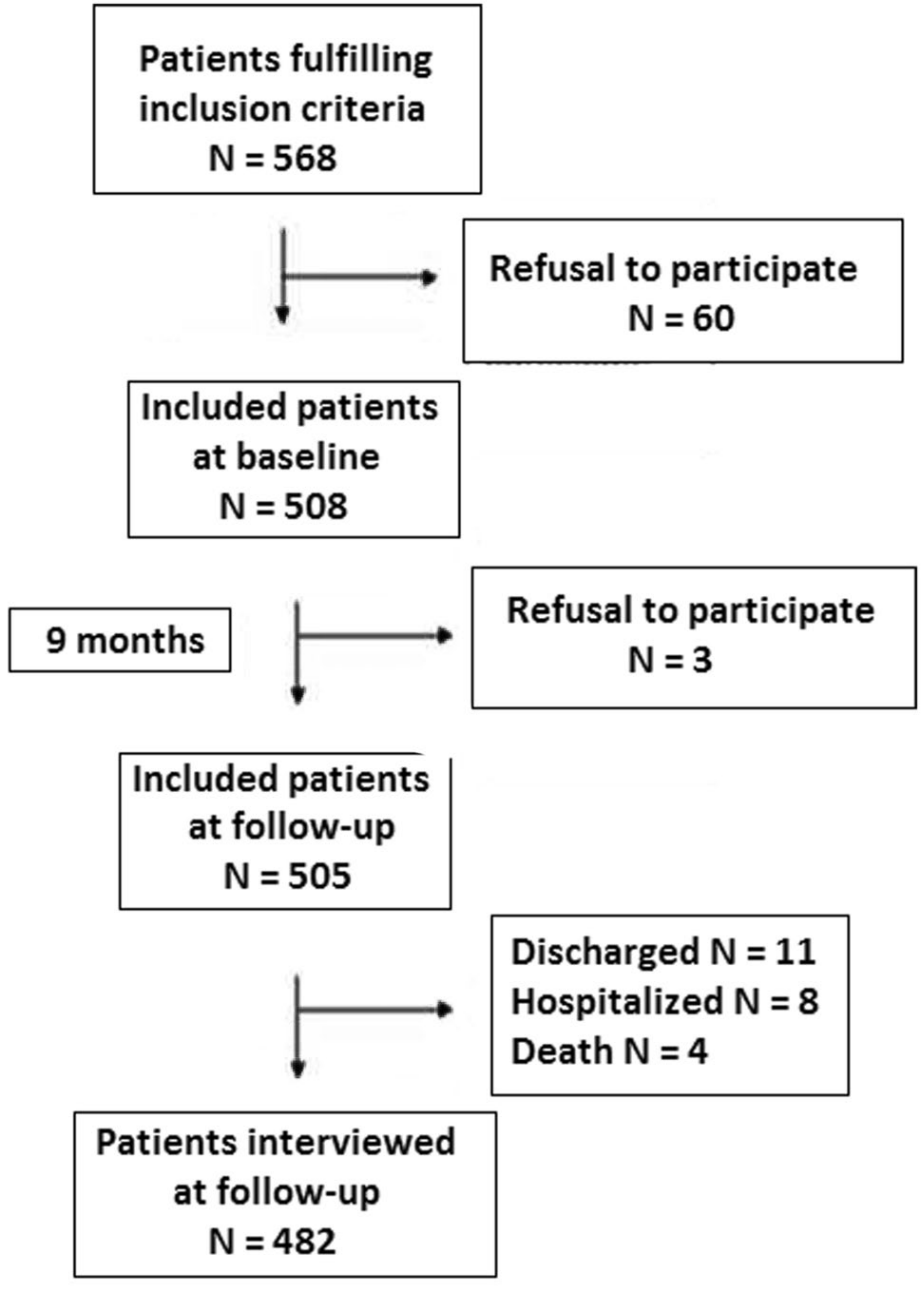
Flow chart of the study population

A total of 508 patients met the inclusion criteria and completed the study questionnaires at baseline. The group with a diagnosis of Schizophrenia included 241 patients, while the group with depression included 267 patients. However, 26 participants were lost to follow-up, so that data at 9 months of follow-up was available for the remaining 482 patients (94.9%). Our reported results reflect mainly baseline data unless measures at follow-up showed significant differences. The flow chart of the study population is shown in Fig. 1.

### Sociodemographic data

Salient findings of sociodemographic data are shown in Table 1. The mean (SD) age was 50.9 (13.2) years (range 18–83). Most patients (51%) were between 50 and 69 years. The mean age for men was 47.9 for men and 52.0 for women. A large percentage of patients (83%) lived with their families. In the distribution by gender, 66% of women lived with their own family, whereas 47% of men lived with their family or by their own (43%).

**Table 1:** Sociodemographic and characteristics of 508 patients at baseline

	Whole sample	Patients with schizophrenia	Patients with depression	
	(*n* = 508)	(*n* = 241)	(*n* = 267)	
**Gender**				
Women	53%	106 (20.8%)	164 (32.29%)	*X*^2^ = 17.7
Men	47%	135 (36.6%)	103 (20.3%)	*p* = 0.00
**Age** (mean ± sd)	50.9 ± 13.2	49.19 ± 12.3	54.33 ± 13.4	*t* = −4.4 *p* = 0.00
**Marital status**				
Single	41.5%	26.8%	14.7%	
Married/stable partner	34.8%	11.1%	23.7%	*X*^2^ = 50.7 *P* = 0.00
Separated/divorced	17.7%	7.3%	10.4%	
Widow	3.9%	1%	2.9%	
Others	2.1%	1.7%	0.4%	
**Living arrangement**				
Own family	44%	14.7%	29.33%	
Family of origin	27.8%	17.4%	10.4%	*X*^2^ = 41.3
Alone	11%	5.3%	5.7%	*P* = 0.00
Protected flat	2.5%	2.1%	0.4%	
Others	14.5%	7.8%	6.6%	

### Clinical and treatment-related findings

To the question: *Do you think you have a mental health problem?* 93.9% of the patients responded affirmatively (95.5 and 93.2% in the schizophrenia and depression group respectively, *X*^2^ = 1.16; *p* = 0.18). When asked what they thought their diagnosis was, responses in order of frequency were: “major depression” by 45.7% of patients (mean age of responders 54.7 [13.1] years), “schizophrenia” by 39% (mean age 49.4 [12.2], and “I don’t know” by 15.7% (mean age 49.5 [14] years). The mean age was significantly higher among patients with depression as compared to the other groups (*p* < 0.001). When participants were asked what psychotropic drugs they were currently taking, they responded: antidepressants (34.7%), antipsychotics (27.4%), antipsychotics plus antidepressants (17.5%), antidepressants and other psychotropic drugs (9.8%), antipsychotics and other psychotropic drugs (5.9%), and antipsychotics with antidepressants and other psychotropic drugs (4.7%).

Most of the patients (93.5%) positively rated the help received by the professionals (95.5 and 92.1% in the schizophrenia and depression group respectively rated the help as “quite” or “a lot”). Regarding the help received by the pharmacological treatment, 92.5 and 75.9% in the schizophrenia and depression group respectively rated the help as “quite” or “a lot”. Each patient was visited by an average of 2.5 different professionals and 96% by a psychiatrist at some point in the last year. Regarding the presence of comorbidities, overall, 67.2% (*n* = 322) of the patients reported suffering from some organic disease (mean 2.2 diseases per patient). This was reported by 76% of patients with depression and 60% of those with schizophrenia. The most common comorbidities were cardiovascular diseases (26%), chronic pain (26%), endocrine diseases (21%), rheumatological disorders (18%), digestive conditions (18%), and respiratory diseases (16%).

**Fig. 2:**
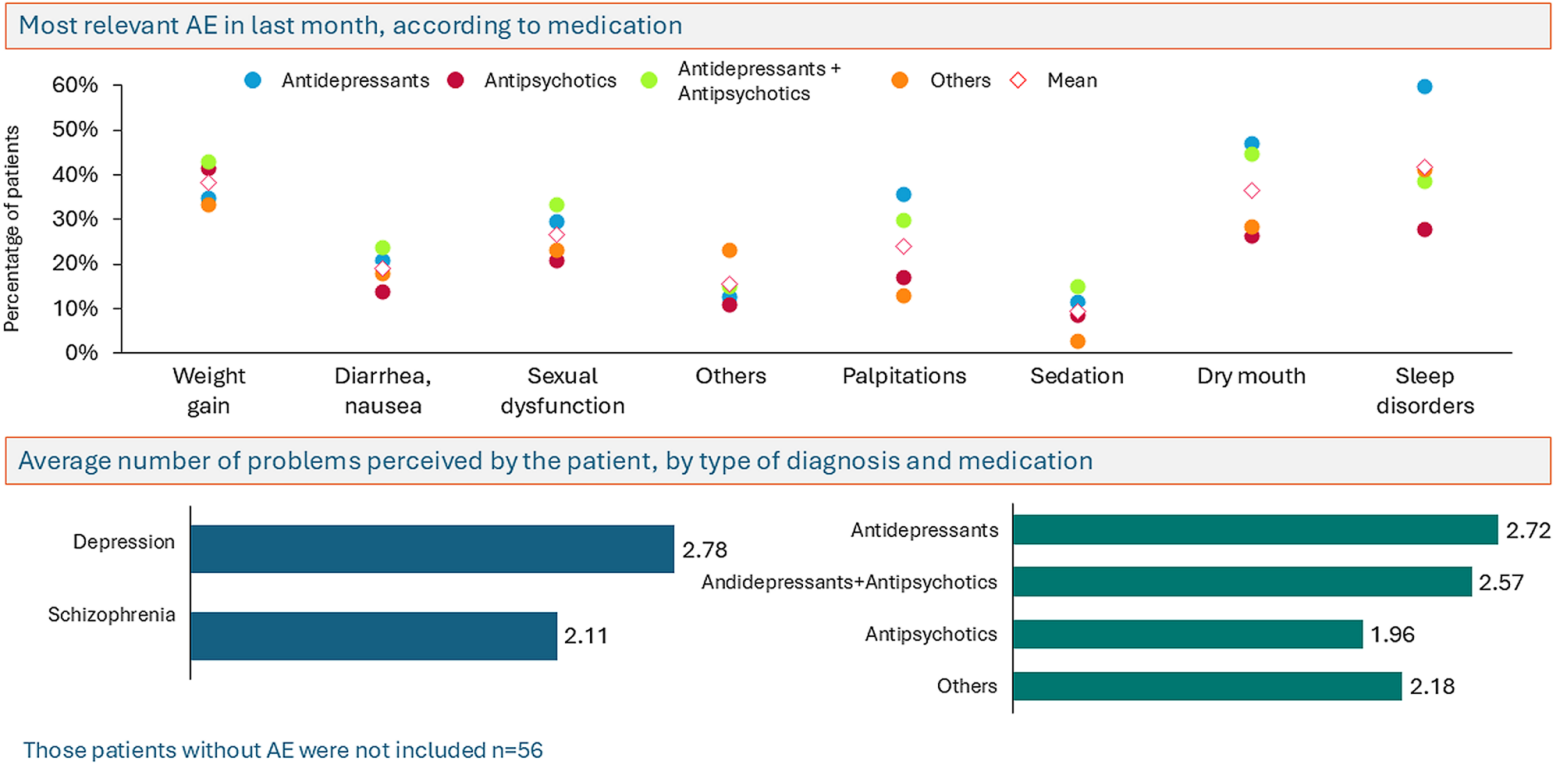
Most relevant adverse events related to medication in the last month referred by patients (56 patients who did not report adverse events were not included)

In relation to the question regarding the number and type of medication-related adverse events, 21.9% of patients reported having no side effects, 19.9% reported one effect, 21.1% two, and 31.7% three or more, being weight gain (34.1%), sleep problems (15%), sexual dysfunction (11.4%), and dry mouth (6.1%) the most common. Patients diagnosed with depression and treated with antidepressants reported a higher frequency of side effects (Fig. 2).

### Patient recovery

When asked if they considered that during the last year their treatment had offered some type of improvement, 83.9% answered affirmatively, with more patients in the schizophrenia group reporting improvement (87.6 and 80.1% in the schizophrenia and depression group respectively, *X*^2^ = 6.9; *p* = 0.03). Regarding the need of urgent visits, no need was reported by 64.8% (67.4 and 62.8% in the schizophrenia and depression group respectively, *X*^2^ = 1.1; *p* = 0.16), but the remaining 35.2% of patients who needed an urgent visit in the past year, 51.7% were attended in an emergency department, 36.5% in any of the participating centers, and 11.8% in the primary care setting. In relation to previous hospitalizations due to their psychiatric disease, 84.4% of patients did not require in-hospital care (83.5 and 85.3% in the schizophrenia and depression group respectively, *X*^2^ = 3.9; *p* = 0.26). 11.8% reported one episode of hospitalization, and 3.8% two or more hospital admissions. To the question: Do you consider that your treatment will offer you some type of improvement in the future, the answers were: no improvement in 3.7 and 6.0% in the schizophrenia and depression group respectively, some improvement (27.3 and 37.2% in the schizophrenia and depression group respectively), mild improvement (21.1 and 27.4% in the schizophrenia and depression group respectively), and much improvement (47.9 and 29.3% in the schizophrenia and depression group respectively) (*X*^2^ = 18.8; *p* = 0.00). Regarding the degree of impact of the mental illness on the present or future well-being/quality of life, the patients’ opinion was none in 10.7 and 7.1% in the schizophrenia and depression group respectively, a bit (28.9 and 16.2% in the schizophrenia and depression group respectively), quite a bit (36.0 and 39.5% in the schizophrenia and depression group respectively), and a lot (24.4 % and 37.2% in the schizophrenia and depression group respectively) (*X*^2^ = 18.2; *p* = 0.00). Currently, how you feel about your mental health problem, the responses were bad (5.4 and 17.7% in the schizophrenia and depression group respectively), regular (24.8 and 47.4% in the schizophrenia and depression group respectively), good (47.9 and 25.6% in the schizophrenia and depression group respectively), and very good (21.9 and 9.4% in the schizophrenia and depression group respectively) (*X*^2^ = 64.2; *p* = 0.00).

### Patients’ attitude towards medication

Patients reported that their degree of adherence to treatment was high (77%) and most of them (80%) believed the medication had a beneficial effect. Globally, the most predominant route of administration of the medication was the oral route (79%), followed by the intramuscular route (6.3%), but in some cases it was a combination of both (14%). The adherence to antipsychotic treatment was higher among those prescribed long-acting medication (94%) compared with antipsychotics taken by the oral route (84%). In relation to some questions concerning adherence to pharmacological treatment, 76.2% stated that they never forget to take their medication (81.4% of patients with schizophrenia and 72.9% of patients with depression, *X*^2^ = 5.1; *p* = 0.01), 94.9% took the medication according to the dosage and schedule prescribed (96.7 and 94.7% in the schizophrenia and depression group respectively, *X*^2^ = 1.1; *p* = 0.19), and 94.7% never discontinued medication by their own in case of improvement (96.3 and 94.7% in the schizophrenia and depression group respectively, *X*^2^ = 0.69; *p* = 0.26). A total of 82.5% of the patients consider that medication was effective in his/her case (88.8 and 77.8% in the schizophrenia and depression group respectively, *X*^2^ = 15.5; *p* = 0.02). The route of administration was mainly the oral route (79.3%) and only 6.3% used intramuscular injections, and 14.4% both oral and intramuscular formulations. Concerns expressed by patients towards medication were greater for antipsychotics (76%) than for antidepressants (67%) (70.7 and 63.5% in the schizophrenia and depression group respectively, *X*^2^ = 2.9; *p* = 0.05).

### Standardized scales

**Fig. 3:**
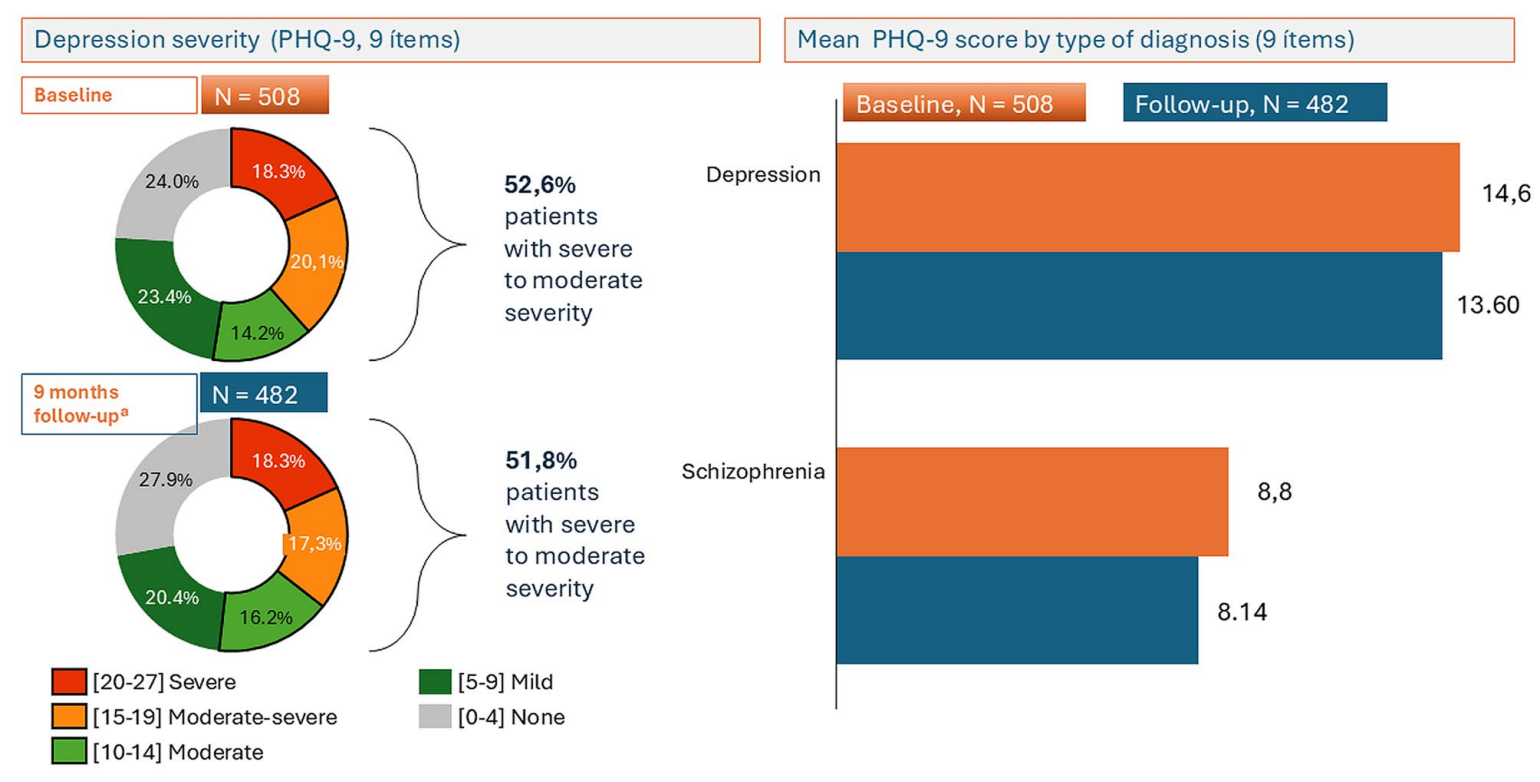
Mean scores of the PHQ-9 questionnaire by depression severity and type of diagnosis. Changes between scores at baseline (*n* = 508) and at 9 months of follow-up (*n* = 482)

Results on the baseline PHQ-9 were severe in 18.3% of patients, moderate-severe in 20.1%, moderate in 14.2%, mild in 23.4%, and none 24% (Fig. 3). Depressed patients showed both at baseline and at follow up significantly more depressive symptoms than the group with schizophrenia (*F* = 66.4; *p* = 0.000) (Table 2). In other words, the effect of treatment group on depressive symptoms across time was statistically significant. In the depressed group, no significant differences in mean PHQ-9 scores between baseline and after 9 months of follow-up were found (*t* = 1.3; *p* = 0.18). However, in the schizophrenia group, a significant improvement in depressive symptoms was noted at 9 months follow-up (*t* = 2.6; *p* = 0.009). These results were confirmed in the ANOVA test (*F* = 7.4; *p* = 0.007).

**Table 2:** Standardized scales in both groups at baseline and after 9 months

	Patients with schizophrenia	Patients with depression	
	(n = 241)	(n = 267)	
**PHQ (depressive symptoms)**			Between subjects: *F* = 66.4; *p* = 0.000 Within subjects: *F* = 7.4; *p* = 0.007
Baseline	8.8 ± 6.7	14.6 ± 7.7	
After 9 months	8.1 ± 6.9	13.6 ± 7.8	
**WHODAS (disability)**			Between subjects: *F* = 37.9; *p* = 0.000 Within subjects: *F* = 24.5; *p* = 0.000
Baseline	50.3 ± 31.4	68 ± 34.5	
After 9 months	24.8 ± 10.6	31.06 ± 12.4	
**SF-12 (quality of life)**			Between subjects: *F* = 0.94; *p* = 0.33 Within subjects: *F* = 0.28; *p* = 0.59
Baseline	30.2 ± 3.2	30.1 ± 2.7	
After 9 months	30.4 ± 3.1	30.1 ± 2.5	

**Fig. 4:**
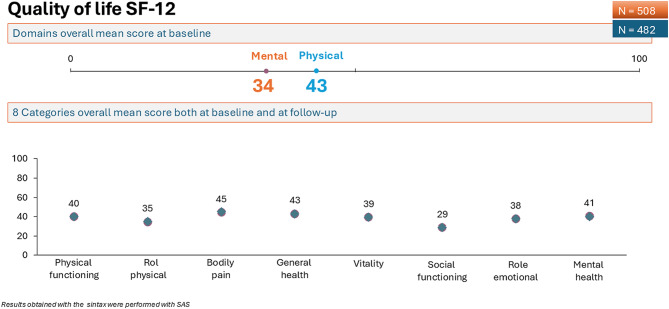
Quality of life measures with the SF-12 questionnaire. Changes between mean scores at baseline (*n* = 508) and at 9-months follow-up (*n* = 482) were not observed

Results obtained with the SF-12 showed a physical score of 43 and a mental score of 34, meaning that patients had a physical quality of life within the normal range but a low mental quality of life. We did not find significant differences within subjects (*F* = 0.28; *p* = 0.59) or between subjects (*F* = 0.94; *p* = 0.33) in the SF-12 scores obtained at baseline and after 9 months of follow-up (Fig. 4 and Table 2) (ANOVA test, General linear model, repeated measures).

Regarding the WHODAS, the average score was 28 (7.1), reflecting impairment in the patients’ functionality. The disability measured by a simple scoring method of WHODAS was extreme (5%), severe (23%), moderate (31%), mild (36%), and none (5%). If we exclude those patients rating “none” in the WHODAS, 95% of our patients reported that their disease had affected their quality of life. Both at baseline and at follow-up, patients with depression reported a significantly higher degree of disability than those with schizophrenia (*F* = 24.5; *p* = 0.000) (Table 2). A significant decrease (less disability) was observed in mean WHODAS scores in both schizophrenia and depression after 9 months (*F* = 37.9; *p* = 0.000) (Table 2). The significant differences found between both groups in the reported disability (WHODAS) and in the depressive symptoms (PHQ-9) supported the authors’ decision to analyze these diagnostic groups separately.

As expected, PHQ-9 scores showed a statistically significant positive correlation with WHODAS scores (*r* = 0.74, *p* < 0.001), reflecting a more severe reported disability in patients with more depressive symptoms. PHQ-9 scores showed a statistically significant negative correlation with SF-12 scores (*r* = −0.17, *p* < 0.001), reflecting the existence of a more impaired quality of life in patients with more depressive symptoms. WHODAS scores were negatively correlated with SF-12 scores (*r* = −0.23, *p* < 0.001) (more impaired quality of life in patients with reported disability).

## Discussion

So far, the implementation of PROMs in routine clinical practice has been hampered by both the lack of agreement about the scales used, the low patient’s completion rate, the scarcity of data at follow-up, the heterogeneity of psychiatric diagnoses and the lack of insight [2]. So far, a Cochrane review [10] did not find sufficient evidence to support the use of routine outcome monitoring using PROMs in mental disorders. Other systematic reviews performed in several mental disorders [5–7] have concluded that there is no evidence for sufficient content validity regarding the PROMs “quality of life” as the patient insight into their illness may vary across psychiatric disorders [10]. To overcome this limitation, several studies have included patients with depression [20–24] and schizophrenia [22–25] or have analyzed both diagnostic groups separately.

This is the first study comparing PROMs variables in a large sample of outpatients with schizophrenia or depression. Our present study follows the ICHOM approach [4] of including physical functioning and treatment expectancy, and variables that are not often included in other PROMS studies, such as social functioning, patient satisfaction and quality of life and achievement of clinical goals [13]. Our study also includes the Cochrane recommendation [9] of a follow-up longer than 6 months. This study provides the subjective perspective of patients with depression and schizophrenia, with stabilized conditions who voluntarily agreed to participate in the study. Most patients were followed for a period of 9 months, so that study instruments could be completed at baseline and at follow-up. Both characteristics of a population of about 500 patients and a length of follow-up of 9 months are salient strengths of the study.

The mean age was practically 51 years, which can be considered representative of the patients who follow treatment at outpatient level. In general, women were older than men, probably due to the greater prevalence of female patients with the diagnosis of major depression disorder. Almost half of the patients (51%) were in the age range of 50–69 years, which is also consistent with the population’s age segment attended in these centers. Most (76.3%) patients were single or married/stable couple and lived with their families (83%). In the distribution by gender, women mostly lived with their own family while men lived with their parents or family of origin. The high percentage of comorbid conditions as reported by patients is an interesting finding, which was also higher in patients with depression compared to schizophrenia. Cardiovascular diseases, musculoskeletal complaints, and chronic pain accounted for 79% of comorbid disorders.

Most patients had a good insight of having a mental health problem, both for major depression and schizophrenia, with an older mean age among patients who recognized having a major depression as compared with patients having schizophrenia (53.7 vs. 48.6 years). Patients who answered that they did not have a mental health problem (6.1%) probably did so because they considered that an affirmative answer might have a stigmatizing meaning. However, a striking finding was a percentage of 15.7% of patients who reported that they did not know the diagnosis of their mental illness.

The highest prevalence of major depression corresponds to the highest prescription of antidepressant drugs, followed by the diagnosis of schizophrenia with the prescription of antipsychotics. We observed that there was a discrepancy between the psychiatric diagnosis and the medication reported by patients, which may be explained by the fact that these patients can take combinations of antipsychotics plus antidepressants and other psychotropic drugs. It should be noted that many patients knew the trade name of the medication they were taking but were totally unaware of the type of psychotropic drug it was. It is important to highlight that most patients positively valued the help of the team of professionals (psychiatrist, psychologist, nursing or social worker) who cared for them, as well as the psychopharmacological treatment they were taking. Regarding adverse events, about 22% of patients said they had no side effects from the medication, or 1 or 2 adverse effects (41%). It is likely that the good tolerability of the medication together with its effectiveness would explain the positive evaluation that patients make of their pharmacological treatment. In the analysis by diagnosis, patients with depression reported a higher frequency of adverse events, which could be justified by a better cognitive performance compared to patients diagnosed with schizophrenia receiving antipsychotics. However, when the question was what level of concern the medication generates, antipsychotic drugs appeared in first place as compared to antidepressants.

Regarding the benefits of the treatment, patients confirmed that in the last year it had represented an improvement for them and in very few cases they had required a visit to an emergency department/urgent visit or a psychiatric hospitalization. Many patients thought they would have mild or much improvement in the future.

In relation to the impact of the illness on the patients’ future well-being/quality of life, most of them rated the impact as “quite a lot”. At the time of the interview and regarding their feelings about their mental health, few participants considered it bad, whereas most patients considered it to be on the average and good or very good. Based on the aforementioned information, it is not surprising that the patients’ attitude towards the medication was positive, with good adherence, and that the majority felt that it had a positive effect on their health.

When comparing the route of administration in patients with a diagnosis of schizophrenia and being treated with long-acting injectable drugs, the level of adherence was higher in this subgroup compared to the oral route. This advantage of long-acting antipsychotic medications is consistent with data reported in other studies [26, 27]. A positive and somewhat unexpected finding was that patients reported taking the medication according to the schedule indicated by their psychiatrist and that they did not stop treatment on their own.

Regarding the standardized scales used as PROMs, 52.6% of our patients showed at baseline a certain level of depression severity assessed with the PHQ-9, which was maintained in the follow-up at 9 months. As expected, patients with a diagnosis of major depression presented higher scores than patients with schizophrenia both at baseline and at follow-up. In our study, depressed patients did not show an improvement of depressive symptoms at follow up. These results are in line with those reported in the study by Steig et al. [21] performed in 171 depressed patients and in another study performed in 629 veterans with depression using the PHQ-9 [20]. Some authors have questioned the usefulness of the PHQ-9 as a depression outcome-based quality indicator due to low sensitivity at follow-up [20]. Also, PHQ-9 outcomes might be confounded by treatment non-adherence when patient’s symptoms improve, a fact that may limit its value. However, in the study by Obarious et al. [4] using a Delphi technique and including the same demographic, clinical and PROMS variables as in our study, the authors propose the PHQ-9 to assess depressive symptoms burden. McKenzie et al. [13] also using a Delphi technique recommend the use of PHQ-9 to measure depressive symptoms and suicidal ideation/behavior and the WHODAS for global and social functioning both at baseline and at follow-up. Our finding of a significant correlation between PHQ, WHODAS and SF-12 scores confirms the validity of this instrument.

In the SF-12 HRQoL questionnaire, both at baseline and at follow-up, physical-related domains were within the normal range despite the high rate of comorbidity, but in the emotional-related domains, most patients considered that their mental illness seriously affected their quality of life. However, no differences were noted in QL between or within our study groups. Our results do not agree with three previous studies [22–24] reporting worse quality of life in patients with depression as compared with schizophrenia. The authors conclude that lack of insight in the patients with schizophrenia and the presence of anhedonia, decreased motivation, low energy level and pessimistic thoughts in the group with depression may account for their results. So far, there has been some controversy regarding the increasingly widespread use of quality of life (QL) outcomes in mental health. Accordingly, it has been proposed that the effect of health care should not be measured by the change in QL alone, but QL should be included alongside other recovery measures.

In the WHODAS 2.0 scale, the average score was 28, which reflects that patient functionality is affected over time. There were no differences between baseline and follow-up in the simple score of difficulties due to health conditions or in the global scores based on simple score. If the ratings of extreme, severe, and moderate were compared with rating of mild or none, a significant level of disability was present. As expected, the group with depression reported a significantly degree of disability than the schizophrenia group, which is in line with our finding of depressed patients reporting more side effects.

This study has some limitations, particularly the representativeness of the study population. Findings obtained in the cohort of patients diagnosed with major depression and schizophrenia under treatment in the nine participating mental health centers are illustrative of the patients’ opinions and values regarding their diseases but cannot be generalized to other mental illnesses or outpatients under treatment in other settings. On the other hand, this is the first study in which two large groups with different psychiatric diagnosis are included, and therefore the reported PROMs or patient generated outcome measures aim to fill in the gap in the existing literature.

## Conclusions

The results of this study are relevant for both patients and clinicians. PROMs have the potential to include the patient´s voice in clinical routine measurement systems, in the evaluation of quality of care, service management and policy making in mental health. Several outcomes considered relevant such as efficacy of medication, side effects, functioning, and HRQoL, would be relatively easy to incorporate in outcome assessment for depression or schizophrenia, as validated questionnaires are available. The evidence of this study can be used to inform clinicians, patients and families about the results obtained and offer the possibility to develop improvement in care and integrate PROMs into routine clinical practice in mental health research.

## Data Availability

Available upon request.

## Abbreviations

ICHOM: International Consortium for Health Outcomes Measurement
PHQ-9: Patient Health Questionnaire
SF-12: Short Form Health Survey (SF-12)
WHODAS 2.0: World Health Organization Disability Assessment Schedule 2.0 (WHODAS 2.0)
